# Children’s Educational Outcomes and Persistence and Severity of Household Food Insecurity in India: Longitudinal Evidence from Young Lives

**DOI:** 10.1016/j.tjnut.2023.02.008

**Published:** 2023-02-11

**Authors:** Thomas Lemma Argaw, Jasmine Fledderjohann, Elisabetta Aurino, Sukumar Vellakkal

**Affiliations:** 1 Department of Sociology, Lancaster University, Lancaster, United Kingdom; 2 School of Economics, University of Barcelona, Barcelona, Spain; 3 Department of Economic Sciences, Indian Institute of Technology Kanpur, Kanpur, India

**Keywords:** children and adolescents, educational outcomes, India, learning, persistence food insecurity, severe food insecurity

## Abstract

**Background:**

Food insecurity is a pressing global challenge with far-reaching consequences for health and well-being. However, little attention has focused specifically on the experiences of children and adolescents over the age of 5 y in food insecure households.

**Objectives:**

We examine whether the persistence and severity of household food insecurity are negatively associated with children’s educational outcomes.

**Methods:**

We used data for the younger cohort of the longitudinal Young Lives data from rounds 3 (2009), 4 (2013), and 5 (2016), when children were aged 8 y, 12 y, and 15 y, respectively. Drawing on the Household Food Insecurity and Access Scale, we used descriptive statistics, graphical analysis, and multilevel regressions to document how the persistence and severity of household food insecurity are associated with children’s educational outcomes (years of education, maths, and vocabulary [PPVT] test scores). We controlled for potentially confounding sociodemographic characteristics, including children’s own baseline grade attained and test scores in “value-added” models, to provide robust estimates of household food insecurity in predicting children’s educational outcomes.

**Results:**

Household food insecurity generally declined between 2009 and 2016. Fewer than 50% of households were food secure across the 3 rounds of data we examined. Our robust, multivariate, value-added models show that the persistence and severity of food insecurity are negatively associated with all 3 children’s educational outcomes we examined.

**Conclusions:**

We add to a small but growing literature exploring how household food insecurity is associated with children’s educational outcomes in the Global South. Our findings on severity of food insecurity highlight the importance of understanding food insecurity along the severity continuum rather than as a dichotomous state, as previously done in existing literature. Addressing household food insecurity in childhood and adolescence may be a key factor to improve children’s educational outcomes.

## Introduction

Food insecurity exists when people do not have consistent physical, social, and economic access to enough safe and nutritious food to support a healthy life [1]. Extant literature has documented the physical health and psychosocial consequences of undernutrition in India, and has shown factors such as gender, family characteristics, genetics, environmental characteristics (including in utero environment), morbidity, and a broader context of rising food prices are associated with increased risk of undernutrition and its sequelae [[2], [3], [4], [5], [6], [7], [8], [9], [10], [11]]. However, food insecurity may affect children well before undernutrition begins to manifest.

Despite this, little attention has focused on children’s experiences of household food insecurity. Moreover, existing literature mostly focuses on children under the age of 5, leaving older children and adolescents comparatively overlooked [12]. In this paper, we draw on longitudinal data from 2 states in India to fill these gaps by documenting associations between educational outcomes and persistence and severity of food insecurity for children aged 8 to 15 y.

### Food insecurity and education in India

Ensuring food security is a core component of poverty eradication strategies in India [13]. In 2016, 38% of Indian children aged less than 5 y were stunted (low height-for-age) and 36% were underweight (low weight-for-age) [14]. Between 2018 and 2020, 15.3% of the Indian population was undernourished. Calculated by FAO, undernourishment indicates a person regularly eats less than the daily minimum calorie requirements (2100 kcal/d for adults). We present undernourishment rather than food insecurity figures here because food insecurity prevalence figures for India are not provided in FAO’s annual report. However, where both undernourishment and food insecurity figures are presented in the report, the prevalence of undernourishment is frequently substantially lower than the prevalence of moderate or severe food insecurity. For instance, in FAO’s 2021 State of Food Insecurity report [1], there is no country in South Asia in which both figures are reported where undernourishment prevalence exceeds moderate or severe food insecurity prevalence. During 2018 to 2020 in the Southern Asia region, which includes India, while the prevalence of undernourishment was 14.1%, the prevalence of moderate or severe food insecurity was 38.7%. There was also a high prevalence in 2020 of wasting (17.3%) and stunting (30.9%) among children under the age 5 y [1]. Data from 2016 to 2018 show 28.4% of Indian young people aged 10 to 19 y are anemic [15], and 24% are thin according to their BMI [16]. Though food insecurity and malnutrition are not synonymous, they are strongly correlated; high rates of malnutrition are suggestive of widespread food insecurity.

Since India’s 2009 Right to Education Act, access to education to Grade (class) 8 is mandatory for children aged 6 to 14 y [17]. The Indian Government has aimed to improve equitable access and learning outcomes via the national school feeding program, the Mid-Day-Meal Scheme, which provides children in government schools with a free cooked meal. This program has had positive outcomes for children’s learning [18, 19], including by mitigating the effects of early shocks on preschool nutritional status [20].

Despite these efforts, national and international discussions have documented Indian children’s underperformance on key learning benchmarks compared with other middle-income countries [[21], [22], [23], [24], [25]]. How children’s experiences of food insecurity at home can shape learning is frequently missing from the debate, despite recent robust evidence that food insecurity from age 5 y is negatively associated with Indian children’s test scores at age 12 y [26]. In light of the high prevalence of food insecurity in India, further attention to links between household food insecurity and educational outcomes is warranted.

### Conceptual links between food insecurity and educational outcomes

**FIGURE 1:**
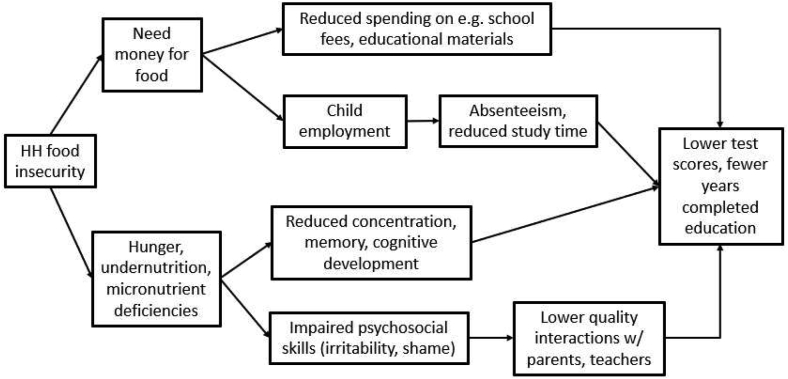
Conceptual model of pathways of the association of food insecurity and children’s educational outcomes.

Figure 1 details our conceptual framework adapted from Fram et al. [27] and Aurino et al. [26]. Importantly, more than 1 pathway may apply in some households. Although not all possible pathways are drawn here, the framework provides an illustrative overview of potential associations between household food insecurity and children’s educational outcomes.

In the pathway at the top of Figure 1, food insecure households need money for food, and may consider trade-offs between spending on food and other household expenditures—in this case, expenditures on children’s educational needs, including school fees, uniforms, study supplies, etc. Lower income and food insecurity have shown to be significantly related to worse educational outcomes for children as parents may limit learning-related expenditures (for example, purchase of books) to reduce their total expenditure [28, 29].

The next pathway in Figure 1 shows food insecure households may rely on children’s employment to meet basic needs. Employed children may have less time to study and, in some cases, may miss school or drop out entirely [[30], [31], [32]]. Food insecure households face higher opportunity costs of sending children, who can make meaningful contributions to household subsistence, to school [33, 34]. This does not imply, however, that children are necessarily passive actors in such decisions; children sometimes actively choose to take up employment opportunities to support their households [35].

In the third pathway, children in food insecure households may experience hunger, undernutrition, and micronutrient deficiencies, impairing their cognitive abilities and development. Food insecure households may compromise the quality, frequency, and portion sizes of food [35, 36], potentially negatively influencing children’s physical growth, cognitive development, test scores, grade completion, and school attendance [31, [36], [37], [38], [39], [40], [41]].

In the final pathway in Figure 1, hunger and undernutrition may impact both parents’ and children’s psychosocial outcomes, influencing interactions within the household and with teachers and peers. Managing limited financial resources can be stressful and anxiety-provoking, impacting parents’ emotional well-being and parenting styles (for example, harsher discipline), which can be detrimental for children’s development [28, 42, 43]. Among children and adolescents, food insecurity is associated with distress, withdrawal, and poorer psychosocial skills [26, 38, 44, 45], and with lower subjective well-being [39, 46]. Academic performance may be influenced by the ability of children to handle emotion and focus their attention [47].

### Study aims

We build on the small number of studies that have examined food insecurity and education in India and other settings in the Global South using longitudinal data, extending beyond what is currently known in several ways. For example, Aurino and colleagues [26] looked at the timing and persistence of food insecurity and learning outcomes at age 12 y, but did not include severity of food insecurity in their analysis, and did not consider older adolescents. Our analysis adds to this literature by examining educational outcomes and food insecurity concurrently across 3 rounds of data, considering the severity of food insecurity, and extending the analysis beyond age 12 y. We focus on the following research questions:

1. Following the same group of children over time, (how) has the severity of household food insecurity changed?
2. (How) is the persistence of food insecurity associated with children’s educational outcomes?
3. (How) is the severity of food insecurity associated with children’s educational outcomes?

## Data and Methods

### Data

We analyze the secondary Young Lives (YL) survey data (for full details of the YL study see Barnett et al. [48]). This rich longitudinal data were collected every ∼3 y since 2002 from households in Ethiopia, India (Andhra Pradesh and Telangana), Peru, and Vietnam. The survey followed 2 cohorts in each country. The younger cohort children (*n* = ∼2000) were aged around 1 y in round 1, and the older cohort children (*n* = ∼1000) were aged around 8 y in round 1. Data were collected at individual, household, and community levels, with variables including sociodemographics and a wide range of variables relating to poverty, health, and well-being. We analyzed the younger cohort data from India, collected across rounds 3 (2009), 4 (2013), and 5 (2016), because these are the rounds in which household food insecurity was measured using a consistent and comparable scale. We do not use older cohort data because part of the sample has moved out of its household in the years in which the food insecurity scales have been collected. The attrition rate in the YL countries is very small [48, 49]. Between rounds 1 and 5, attrition was 3.7% for the Indian younger cohort. If we consider the 3 rounds that we used for this analysis, attrition is 1.4%. Barnett et al. [48] have found that this very low attrition rate in the YL data is unlikely to bias inferences.

### Dependent variables

Our dependent variables are years of education completed, maths assessment score, and the Peabody Picture Vocabulary Test (PPVT) score. These were collected in rounds 3 (2009), 4 (2013), and 5 (2016) of the YL data. The maths and vocabulary tests were developed and validated by child development experts. These tests are administered using the local language and by displaying an item for which the child tells the name of the item or gives the answers to a maths quiz. Therefore, maths and PPVT scores are calculated as the total number of words or maths questions a child has answered correctly. We standardized the maths and PPVT scores to have a mean value of 0 and a standard deviation of 1. Standardization is common practice in education studies to facilitate intuitive interpretations of changes in a variable of interest [49, 50]. Although our models used the standardized scores, we provide descriptive statistics for the raw scores for reference in Table 1 in the next section.

**TABLE 1:** Descriptive statistics for the variables used in this study, younger cohort, Young Lives dataset

	Round 3 (2009)			Round 4 (2013)			Round 5 (2016)		
	*n*	Mean	SD	*n*	Mean	SD	*n*	Mean	SD
Years of education completed (y)	1841	1.68	0.97	1890	5.43	1.30	1870	8.08	2.00
Maths test score (count)	1881	12.01	6.44	1836	12.74	6.62	1812	10.30	5.13
PPVT test score (count)	1878	58.35	30.35	1880	43.02	7.84	1858	47.33	7.90
Food insecurity variables									
Persistence food insecurity (ratio)	1908	0.27	0.30	1890	0.27	0.30	1870	0.27	0.30
*Severity of food insecurity (%)*									
Food secure	1908	28.35	45.08	1890	48.52	49.99	1866	43.52	49.59
Mild food insecurity	1908	41.30	49.25	1890	27.57	44.70	1866	30.71	46.14
Moderate/severe food insecurity	1908	30.35	45.99	1890	23.92	42.67	1866	25.78	43.75
Child characteristics									
Male (%)	1908	53.56	49.89	1890	53.81	49.87	1870	53.90	49.86
Child age (mo)	1908	95.39	3.83	1890	143.79	3.81	1870	191.69	4.76
Birth order (number)	1908	1.75	1.03	1890	1.75	1.02	1870	1.76	1.02
Sibling (number)	1908	2.45	0.93	1890	2.55	1.02	1870	2.30	0.87
Child attends private school (%)	1908	43.82	49.63	1890	32.33	46.79	1870	32.14	46.71
Household characteristics									
Household head age (y)	1904	38.60	9.15	1890	41.25	7.95	1870	43.60	7.45
Household heads completed primary education (%)	1908	40.78	49.15	1890	40.74	49.15	1870	40.75	49.15
Household lives in an urban area (%)	1908	25.47	43.58	1890	25.29	43.48	1870	24.92	43.27
*Household wealth profile (%)*									
Lowest wealth tercile	1906	49.53	50.01	1890	29.95	45.81	1870	21.44	41.05
Middle wealth tercile	1906	28.80	45.30	1890	37.83	48.51	1870	32.62	46.89
Top wealth tercile	1906	21.67	41.21	1890	32.22	46.75	1870	45.94	49.85
*Household head caste/tribe (%)*									
Scheduled caste	1908	18.45	38.80	1890	18.36	38.73	1870	18.50	38.84
Scheduled tribe	1908	14.94	35.65	1890	14.97	35.69	1870	14.97	35.69
Backward caste	1908	46.49	49.89	1890	46.61	49.90	1870	46.47	49.89
Other category	1908	20.13	40.10	1890	20.05	40.05	1870	20.05	40.05

### Independent variables

Our key independent variable of interest is household food insecurity. In milder forms, food insecurity can involve experiences such as worries about food, whereas moderate or severe forms may involve cutting back on food, skipping meals, or going for entire days without food [51]. Such experiences may be transitory, occurring over a short period, or more persistent, occurring over an extended period or at frequent intervals. To measure household food insecurity, we used the experience-based food insecurity measurement scale, the Household Food Insecurity Access Scale (HFIAS). The HFIAS is composed of binary items about: worries about food shortage, eating undesired foods, eating less, reducing number of meals, and not eating the whole day and night, whether any household member was not able to eat desired foods because of lack of money, whether household members had to eat limited range of food, whether there was no food to eat because of lack of money, and whether household members had to go to bed hungry.

Unlike the more typical HFIAS 30-d recall period, the HFIAS in YL is collected based on a 12-mo recall period. We coded a categorical variable for severity of food insecurity (not food insecure, mild, moderate, or severe) following the standard HFIAS procedure [52]. However, because the prevalence of severe food insecurity was low, resulting in a small cell size (3.65%; *n* = 207), we merged the severe and moderate (23.02%; *n* = 1305) categories, resulting in a collapsed moderate or severe category (26.68%; *n* = 1512). The remaining categories (mildly food insecure, not food insecure) were unchanged. We computed persistence of food insecurity as ratio of the number of rounds a child’s household has been food insecure to the number of rounds the child has been surveyed. For children who were enrolled in school in all 3 rounds, this variable is the number of those rounds spent in which the child was in food insecure household in the numerator divided by 3. This variable has no standard unit and ranges between 0 and 1, with 0 indicating that the child’s household was never food insecure, 1 indicating that the child's household was persistently food insecure across the observed rounds, and a value between 0 and 1 indicating transitory food insecurity.

Following extant literature from India highlighting socioeconomic status, sociodemographic characteristics, and household composition as important correlates of food insecurity and dietary patterns [[53], [54], [55], [56], [57], [68]], we controlled for several possible confounders in our analysis. Household level correlates included household head’s age, education (primary completed compared with no primary education completed), and household head caste or tribe (scheduled caste or tribe; scheduled tribe; backward caste or tribe; other), household place of residence (rural compared with urban), and wealth tercile (low; middle; top). Three categories of wealth were calculated as lowest, middle, and top wealth terciles from the YL wealth index, composed of housing quality, access to services, and ownership of consumer durables [58]. Child-level characteristics included child sex, age (in months), number of siblings, birth order, and child’s school type (private compared with government).

**FIGURE 2:**
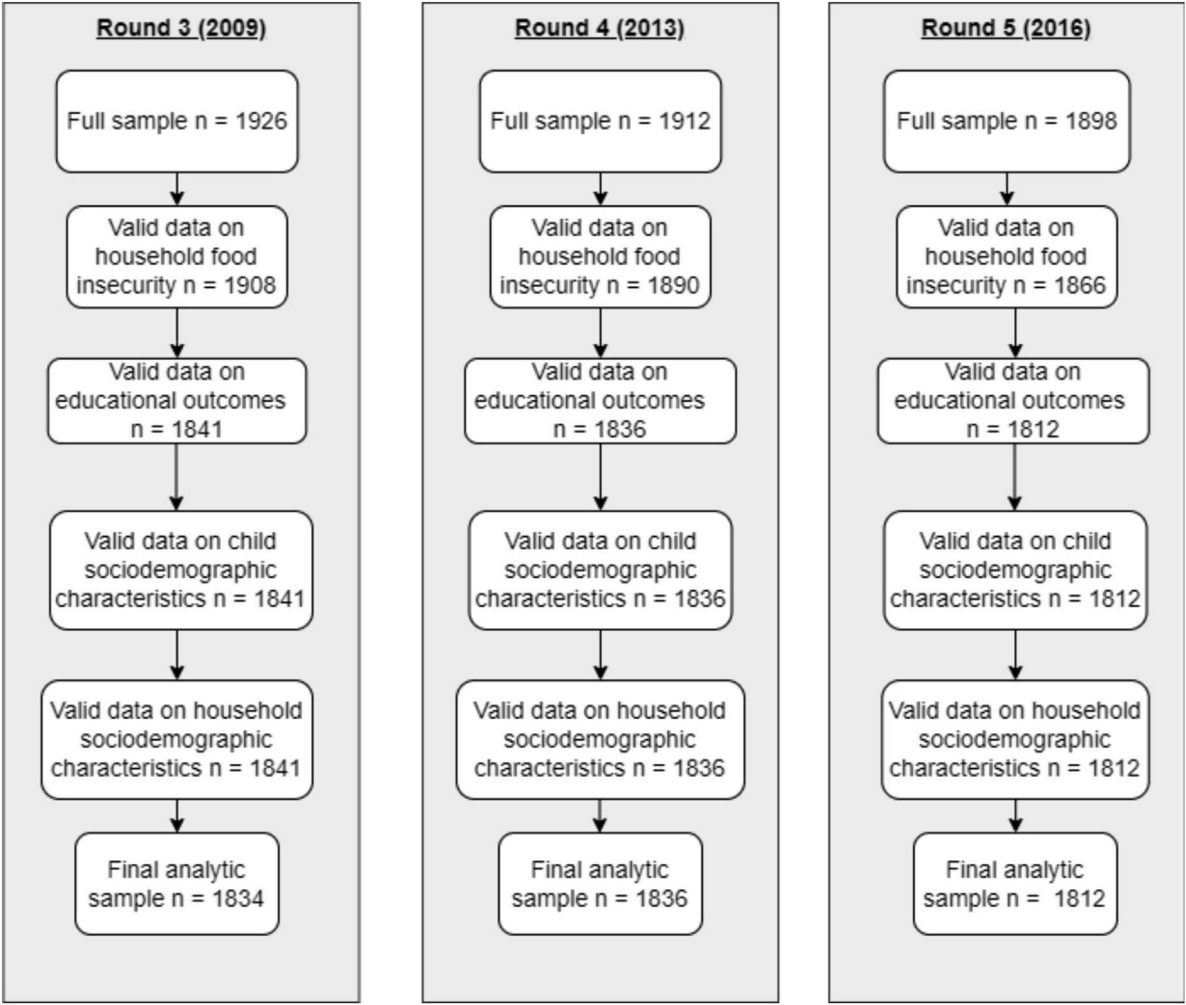
Flowchart showing the construction of the sample used for regression analysis.

In Figure 2, we have presented the data cleaning procedure followed and the final analytic sample for each round. Our data are 96% balanced surveying children in each of the 3 rounds. We have used all the data available in each round in our study. As can be seen, we have a few data points missing, arising from data collection in each round and across rounds. Further details on data missingness related to the variables we have used in our analysis can be found in Table 1.

### Analytic strategy

To account for clustering of children in households within communities, we applied multilevel mixed-effects random intercept linear regression models. However, unobserved child and parental heterogeneity may lead to the problem of endogeneity arising from omitted variables. There could also be measurement bias in the educational outcomes we considered. We therefore used value-added models of achievement [59, 60], which control for baseline scores—in this case each of our educational outcomes (level of education completed, PPVT, and maths scores) from round 2 in respective models. Value-added models are a robust approach to address unobserved heterogeneity and measurement bias [61]. Despite our use of value-added models and the inclusion of a set of child and household-level sociodemographics, our results should only be interpreted as robust associations and not as causal.

To assess the extent to which the association between household food insecurity and educational outcomes is explained by sociodemographic characteristics, we included child and household-level covariates extant literature has been identified as salient. Model 1 includes child characteristics and baseline education and learning outcomes from round 2. In Model 2, household sociodemographic indicators are also considered. Model 2 represents our most robust model, which accounts for our full set of potential confounders in modeling the association between food insecurity and educational outcomes. All analyses were completed using Stata v.16.

Equation 1 gives a multilevel mixed-effects random intercept linear model:

(1) $${Ed}_{ihc,t}=\beta_{0}+Z_{ihc,t}^{\prime }\beta_{1}+X_{hc,t}^{\prime }\beta_{2}+\upsilon_{ih}+\upsilon_{c}+\varepsilon_{ihc}$$

${Ed}_{ihc}$ is the dependent variable (years of education completed, standardized PPVT, and standardized maths scores). $Z_{ihc}^{\prime }\text{,}$refers to child characteristics in household h including baseline scores, $X_{hc}^{\prime }$ contains sociodemographic indicators, including persistence and severity of household food insecurity, $\upsilon_{ih}$ and $\upsilon_{c}$are the random effects at the household (the first) level and households within community (the second level), and $\varepsilon_{ihc}$, refers to the unobserved heterogeneity. We used the model fit criterion of intraclass correlation (ICC) and the likelihood ratio test comparing multilevel random intercept models against an ordinary linear regression model to test model fit. $Z_{ihc,t}^{\prime }$ and $X_{hc,t}^{\prime }$comprise the fixed component of the multilevel model, whereas $\upsilon_{ih},\upsilon_{c},and\;\varepsilon_{ihc}$ make up the random component. Unobserved heterogeneities over and above the ones we have controlled for will be accounted for by the random components of $\upsilon_{ih},\upsilon_{c},and\;\varepsilon_{ihc}$.

## Results

### Descriptive statistics

The percentage of food secure households increased overall from 28.5% in 2009 to 43.5% in 2016. Mild food insecurity followed a U-shaped trend, decreasing from 41.1% in 2009 to 27.9% in 2013 and back up to 30.7% in 2016. A similar trend was observed for moderate or severe food insecurity, albeit with small changes in magnitude from 30.4% in round 3 to 24% in round 4, then slightly rising back to 25.8% in round 5 (Pearson χ^2^ = 173.97, *p* < 0.000).

Children’s years of completed education increased roughly linearly across rounds. Mean raw maths scores followed a U-shaped path, whereas mean PPVT scores slightly declined over time.

Boys made up ∼53.7% of the study sample overall. On average, children were not firstborn, but were still of low birth order. Children had just over 2 siblings on average. Although 36.76% of children were enrolled in private school overall, there was a notable drop from 42.97% in 2009 down to around 32% in subsequent rounds. The household head’s age also increased linearly, and around 41% of household heads had completed primary education across rounds. There was little variation in the place of residence, with roughly one-quarter of households living in urban areas across rounds. The percentage of households in the lowest wealth tercile declined from 49.16% to 21.39% from 2009 to 2016, whereas the percent in the top wealth tercile increased over the study period, from 20.42% to 43.21%. The percent of households in the middle wealth tercile increased to 35.40% in 2016 from 30.42% in 2009. In terms of caste or tribe, the highest proportion of household heads identified as backward caste or tribe (46.55%), followed by other (20.29%), scheduled caste or tribe (18.18%), and scheduled tribe (15.07%) (Table 1).

### Regression results

Abbreviated results from the estimated multilevel linear regression models are presented in TABLE 2, TABLE 3. Results showing all coefficients are available in Supplemental Tables 1 to 6. Overall, the multilevel models resulted in a good fit, as the intraclass correlation coefficients and the likelihood ratio tests showed. From the intraclass correlation, 7% of the variation in years of completed education and PPVT, and 5.54% in maths scores was attributed to the community variation. Up to 51% of the difference in years of completed education and 43% in maths score was accounted for by a combination of community and household differences in the persistence models. The severity models also captured sizable differences, albeit in relatively smaller magnitude. The conservative likelihood ratio test justified the use of the multilevel random intercept model: the models captured community and household differences in educational outcomes considered (*P* < 0.01).

**TABLE 2:** Association of persistence of food insecurity and children’s educational outcomes—young Lives dataset for years 2009, 2013, and 2016

		Years of education completed		PPVT^5^ score		Maths score	
		Model 1	Model 2	Model 1	Model 2	Model 1	Model 2
		b (Se)^2^	b (Se)	b (Se)	b (Se)	b (Se)	b (Se)
	Persistence of food insecurity	−0.31∗∗∗^6^ (0.07)	−0.19∗∗∗ (0.07)	−0.22∗∗∗ (0.04)	−0.15∗∗∗ (0.04)	−0.24∗∗∗ (0.03)	−0.17∗∗∗ (0.03)
Additional variables	Child characteristics^1^	Yes	Yes	Yes	Yes	Yes	Yes
	Baseline years education	Yes	Yes	Yes	Yes	Yes	Yes
	Household characteristics^3^	No	Yes	No	Yes	No	Yes
Intraclass correlations							
Community		8.37%	6.99%	7.90%	7.34%	6.00%	5.48%
Household | community		48.63%	48.84%	16.70%	16.22%	43.97%	43.60%
LR^4^ test vs. linear model chi2(2)		228.26∗∗∗	181.91∗∗∗	175.99∗∗∗	158.13∗∗∗	160.51∗∗∗	139.42∗∗∗

1 Child sex, child age, number of siblings, birth order, whether a child attends in private school, and baseline learning outcome values.

2 Coefficient (Standard error).

3 Household head education (primary complete), household head age, place of residence, wealth tercile (1. Lowest wealth tercile, 2. Middle wealth tercile, 3. Top wealth tercile), and household head caste/tribe. (1. Scheduled caste/tribe, 2. Scheduled tribe, 3. Backward caste, 4. Others category.).

4 Likelihood ratio.

5 Peabody Picture Vocabulary Test.

6 Statistical level of significance at ∗*P* < 0.1, ∗∗*P* < 0.05, ∗∗∗*P* < 0.01.

**TABLE 3:** Association of severity of food insecurity and children’s educational outcome—young Lives dataset for years 2009, 2013, and 2016

		Years of education completed		PPVT^5^ score		Maths score	
		Model 1	Model 2	Model 1	Model 2	Model 1	Model 2
		b (Se)^2^	b (Se)	b (Se)	b (Se)	b (Se)	b (Se)
	Food secure	Reference	Reference	Reference	Reference	Reference	Reference
	Mild	−0.30∗∗∗^6^ (0.05)	−0.25∗∗∗ (0.05)	−0.02 (0.03)	0.01 (0.03)	−0.06∗∗ (0.03)	−0.03 (0.03)
	Moderate/severe	−0.30∗∗∗ (0.05)	−0.22∗∗∗ (0.05)	−0.19∗∗∗ (0.03)	−0.13∗∗∗ (0.03)	−0.19∗∗∗ (0.03)	−0.13∗∗∗ (0.03)
**Additional variables**	Child characteristics^1^	Yes	Yes	Yes	Yes	Yes	Yes
	Baseline education	Yes	Yes	Yes	Yes	Yes	Yes
	Household characteristics^3^	No	Yes	No	Yes	No	Yes
**Intraclass correlations**							
**Community**		8.02%	6.78%	7.89%	7.29%	7.89%	7.29%
**Household | community**		48.62%	48.85%	17.26%	16.60%	17.26%	16.60%
**LR**^4^**test vs. linear model chi2(2)**		219.07∗∗∗	177.65∗∗∗	173.72∗∗∗	154.36∗∗∗	173.72∗∗∗	154.36∗∗∗

1 Child sex, child age, number of siblings, birth order, whether a child attends in private school and baseline learning outcome values.

2 Coefficient (Standard error).

3 Household head education (primary complete), household head age, place of residence, wealth tercile (1. Lowest wealth tercile, 2. Middle wealth tercile, 3. Top wealth tercile), and household head caste/tribe (1. Scheduled caste/tribe, 2. Scheduled tribe, 3. Backward caste, 4. Others category.)

4 Likelihood ratio.

5 Peabody Picture Vocabulary test.

6 Statistical level of significance at ∗*P* < 0.1, ∗∗*P* < 0.05, ∗∗∗*P* < 0.01.

We started by examining the associations between persistence of household food insecurity and educational outcomes (Table 2). Model 1 for years of education completed, which gives the basic value-added model (accounting for children’s characteristics and their baseline years of education at round 2), showed a strong, negative association between persistence of food insecurity and years of education completed (b = −0.31; *P* < 0.01). Although the association weakened with the inclusion of household sociodemographic characteristics in Model 2 (b = −0.19; *P* < 0.01), it remained strong and significant. Model 1 for PPVT scores also showed a strong, negative association between persistence of food insecurity and PPVT scores (b = −0.22; *P* < 0.01) after accounting for children’s characteristics and their baseline scores following the value-added framework. As with years of completed education, this association weakened with the introduction of household sociodemographic characteristics in Model 2 (b = −0.15; *P* < 0.01). A very similar pattern of results is also shown for maths scores.

Examining the associations of the severity of food insecurity with years of education completed shows a strong, negative association. Model 1 showed a strong association between severity of food insecurity with children’s years of education completed and accounting for child sociodemographic characteristics (mild: b = −0.30, *P* < 0.01; moderate or severe: b = −0.30, *P* < 0.01). As Model 2 showed, adding household sociodemographic characteristics only slightly reduced the negative association (mild: b = −0.25, *P* < 0.01; moderate or severe: b = −0.22, *P* < 0.01) between food insecurity and years of education completed. PPVT score showed no significant negative association with mild food insecurity in Models 1 and 2. For maths, the value-added results from Model 1 showed a negative association (b = −0.06, *P* < 0.01) with the inclusion of child sociodemographics. However, this association is attenuated to nonsignificance with the introduction of household sociodemographics in Model 2. Moderate or severe food insecurity, on the other hand, is negatively associated with children’s test scores with the introduction of child and household sociodemographics, as shown in Model 2 (PPVT: b = −0.13, *P* < 0.01; maths: *b* = −0.13, *P* < 0.01) (Table 3).

### Robustness checks

We estimated 2 separate sets of models as a robustness check and found that our results were robust to different specifications. First, to address the possibility that our results were an artifact of the coding of our severity and persistence variables, we fit our models using a dichotomous indicator of food secure compared with food insecure households. The results are included in Supplemental Tables 7 to 9 Second, understanding that the Indian economy was largely volatile during this period, we included round of data collection as an additional explanatory variable in our models. We confirm that the results in both specifications were similar to those presented in our main results, except minor attenuation in the coefficients of the main variables (available upon request).

## Discussion

This study investigated the association of the persistence and severity of household food insecurity with children’s years of completed education and learning (PPVT and maths test scores). To begin, we looked at how the severity of food insecurity at home has changed over time within the cohort. We found that both mild and moderate or severe food insecurity generally declined between 2009 and 2016, though there was a slight increase between 2013 and 2016. However, the percent of households that were food secure never exceeded 50% across the 3 rounds of data that we examined.

Next, we sought to understand how the persistence and severity of food insecurity at home were associated with children’s educational outcomes. Overall, our results show that both the persistence and severity of food insecurity are critically associated with children’s educational outcomes. The magnitude of associations may appear to be small, but they are not negligible. To put the results in context, the average effect of educational interventions in the Global South to improve learning do not often go beyond 0.10 standard deviation mark [62], while most of the associations we found were above this mark.

A novel aspect of our study—compared with much of the extant literature in the context of Global South on food insecurity and education [24, 47]—is to examine the severity of food insecurity. Our findings highlight the importance of understanding food insecurity along the severity continuum. This is consistent with conclusions from Pérez-Escamilla et al. [63]. Their review of the global literature on food insecurity emphasized different levels of severity of food insecurity are associated with different risk factors (overweight, underweight, obesity, etc.), requiring specific policy interventions based on these different levels. Moreover, our results are consistent with previous studies from the Global South [26, 30, 50] and from the US [38, 64], which show negative associations between household food insecurity and children’s cognitive skills.

Finally, we considered whether sociodemographics accounted for the associations we observed between food insecurity and educational outcomes. Previous extant studies have highlighted that sociodemographic variables including household composition [26, 65], type of school [22], socioeconomic position, and caste or tribe [66] are strong predictors of learning outcomes. To this end, we introduced child and household sociodemographic variables as key predictors of educational outcomes in our models. Our models reveal that the persistence and severity of food insecurity continued to predict poorer educational outcomes even after we controlled for additional child and household-level sociodemographic characteristics. These results show that food insecurity during childhood is an important predictor of children’s educational trajectories, with potential repercussions for their labor market, income, and health trajectories.

This study has several strengths and limitations. Our results are based on a longitudinal dataset. By fitting value-added models controlling for baseline test scores, we addressed issues related to unobserved heterogeneity and measurement bias. We also considered the household food insecurity experiences of children aged 8 to 15 y, expanding literature on a “forgotten population” in food insecurity studies [12]. Our study adds to the existing literature by looking at concurrent associations between food insecurity and educational outcomes across an extended period of childhood. In terms of limitations, persistence and severity of food insecurity were measured at the household level, and therefore cannot account for intrahousehold variation in the experience of food insecurity. In particular, YL lacks direct measurement of children’s food insecurity measured through recently developed experiential scales such as by Frongillo et al. [67]. Related to this, we were unable to consider the quantity and quality of food children consumed. Though we have controlled for child and household-level characteristics, measures of children’s own experiences of food insecurity may shed light on issues of intrahousehold disparities in their access to food [66]. Finally, it is possible that we have underestimated the persistence of food insecurity because of measurement error between rounds: the recall window for the HFIAS is 12 mo, but the data were collected roughly every 3 y. Some households that did or did not report food insecurity during any of the observation periods may or may not have experienced food insecurity in intervening years, which would introduce a conservative bias into our estimates on persistence and educational outcomes. We also want to emphasize that future research using the YL data should examine whether these associations hold for the other YL countries.

Our findings can be used as a starting point to identify or potentiate relevant social protection programs with the potential to address children’s experiences of food insecurity at home and improve educational outcomes, such as, for instance, school feeding. We measured food insecurity at the household level. Not all children in food insecure households will themselves experience difficulties accessing enough safe and nutritious food because of the mechanisms of coping and buffering operating within households. Nonetheless, even food secure children in food insecure households will not be fully insulated from budgeting and emotional stress that the household experiences. Therefore, extant social protection programs that can reduce food insecurity may have the additional potential benefit of improving educational outcomes. Children’s health and well-being are important reasons in their own right to invest in addressing household food insecurity, but these programs can also positively enhance learning, as shown in India [18, 19] and elsewhere in the Global South [69]. Investment in children’s food security may also be seen as an investment in human capital development, with potential long-term economic benefits for India’s economic future [70].

In conclusion, we added to a small but growing body of literature exploring how household food insecurity is associated with children’s educational outcomes in India. Our results show that both persistent and severe food insecurity have negative consequences for children; both phenomena are associated with fewer completed years of education and lower maths and PPVT scores. As we indicated in our analytic strategy, we do not aim to make a causal claim here. However, our findings suggest that addressing household food insecurity in childhood and adolescence could potentially improve children’s educational outcomes.

## Author Contributions

The authors’ responsibilities were as follows – ATL, JF, EA, SV: conceived the study; ATL: cleaned and prepared the data; ATL, JF: developed the analytic framework, analyzed, and interpreted the data, and wrote the initial manuscript; all authors contributed to interpreting and contextualizing findings and to manuscript revision. ATL, JF, EA, SV: read and approved the final version.

## Funding

Support for this project was provided by a grant from UKRI through a Future Leaders Fellowship [grant number MR/T021950/1]. Aurino is grateful to the Jacobs Foundation for its support through a Jacobs Foundation research fellowship. We are grateful to Young Lives and the children and families participating to the study, and we appreciate the Young Lives project for making the Young Lives longitudinal dataset publicly available.

## Author disclosures

The authors report no conflicts of interest.

## Data availability

The publicly available Young Lives dataset is used for this analysis. The dataset can be accessed online to registered users through the UK Data Service. https://beta.ukdataservice.ac.uk/datacatalogue/series/series?id=2000060.

## Appendix A Supplementary data

Multimedia component1 Multimedia component1
