# Supplementary material for: Children’s Educational Outcomes and Persistence and Severity of Household Food Insecurity in India: Longitudinal Evidence from Young Lives
Source: J Nutr. 2023 Feb 11;153(4):1101–10. doi: 10.1016/j.tjnut.2023.02.008 (PMC10196702; doi:10.1016/j.tjnut.2023.02.008)

**Argaw Thomas.** Children’s educational outcomes and persistence and severity of household food insecurity in India: Longitudinal evidence from Young Lives.

Supplementary Table 1. Association of persistence of food insecurity and children's years of education completed - Young Lives dataset for years 2009, 2013 and 2016.

|  | Model 1 | | Model 2 | |  |
| --- | --- | --- | --- | --- | --- |
|  | Coefficient | Se^2^ | Coefficient | Se |  |
| Persistence of food insecurity | -0.31***^3^ | (0.07) | -0.19*** | (0.07) |  |
| **Children’s characteristics** |  |  |  |  |  |
| *Child sex* |  |  |  |  |  |
| Girl | Reference |  | Reference |  |  |
| Boy | -0.27*** | (0.04) | -0.26*** | (0.04) |  |
| Child age in months | 0.07*** | (0.00) | 0.07*** | (0.00) |  |
| Birth order | -0.07*** | (0.02) | -0.07*** | (0.02) |  |
| Number of siblings | -0.05** | (0.02) | -0.04* | (0.02) |  |
| Child attends in private school | -0.11** | (0.05) | -0.26*** | (0.05) |  |
| Base education | 0.01 | (0.01) | 0.01 | (0.01) |  |
| **Household characteristics** |  |  |  |  |  |
| Household head age(years) |  |  | 0.00 | (0.00) |  |
| *Household head completed primary education* |  |  |  |  |  |
| No |  |  | Reference |  |  |
| Yes |  |  | 0.16*** | (0.04) |  |
| *Household residence* |  |  |  |  |  |
| Rural |  |  | 0.00 | 0.00 |  |
| Urban |  |  | -0.08 | (0.06) |  |
| *Household wealth status* |  |  |  |  |  |
| Lowest wealth tercile |  |  | Reference |  |  |
| Middle wealth tercile |  |  | 0.17*** | (0.05) |  |
| Top wealth tercile |  |  | 0.36*** | (0.06) |  |
| *Household head caste/tribe* |  |  |  |  |  |
| Scheduled Caste |  |  | Reference |  |  |
| Scheduled Tribe |  |  | -0.24*** | (0.07) |  |
| Backward Classes |  |  | -0.04 | (0.06) |  |
| Other Category |  |  | -0.02 | (0.07) |  |
| Intraclass correlations |  |  |  |  |  |
| Community | 8.37%  48.63%  228.26*** | | 6.99%  48.84%  181.91*** | |  |
| Household \| community |  |  |  |  |  |
| LR^1^ test vs linear model chi2(2) |  |  |  |  |  |
| n | 5592 | | 5592 | |  |

*^1^ Likelihood ratio*

*^2^ Standard error*

*^3^. * p<0.1, ** p<0.05, *** p<0.01*

Supplementary Table 2. Association of persistence of food insecurity and children's maths score - Young Lives dataset for years 2009, 2013 and 2016.

|  | Model 1 | | Model 2 | |  |
| --- | --- | --- | --- | --- | --- |
|  | Coefficient | Se^2^ | Coefficient | Se |  |
| Persistence of food insecurity | -0.24***^3^ | (0.03) | -0.17*** | (0.03) |  |
| **Children’s characteristics** |  |  |  |  |  |
| *Child sex* |  |  |  |  |  |
| Girl | Reference |  | Reference |  |  |
| Boy | 0.03 | (0.02) | 0.03* | (0.02) |  |
| Child age in months | 0.00 | (0.00) | -0.00* | (0.00) |  |
| Birth order | -0.03*** | (0.01) | -0.03*** | (0.01) |  |
| Number of siblings | -0.01 | (0.01) | -0.01 | (0.01) |  |
| Child attends in private school | 0.18*** | (0.02) | 0.11*** | (0.02) |  |
| Base maths score | 0.67*** | (0.01) | 0.66*** | (0.01) |  |
| **Household characteristics** |  |  |  |  |  |
| Household head age(years) |  |  | 0.00 | (0.00) |  |
| *Household head completed primary education* |  |  |  |  |  |
| No |  |  | Reference |  |  |
| Yes |  |  | 0.13*** | (0.02) |  |
| *Household residence* |  |  |  |  |  |
| Rural |  |  | Reference |  |  |
| Urban |  |  | -0.08** | (0.03) |  |
| *Household wealth status* |  |  |  |  |  |
| Lowest wealth tercile |  |  | Reference |  |  |
| Middle wealth tercile |  |  | 0.04 | (0.02) |  |
| Top wealth tercile |  |  | 0.16*** | (0.03) |  |
| *Household head caste/tribe* |  |  |  |  |  |
| Scheduled Caste |  |  | Reference |  |  |
| Scheduled Tribe |  |  | 0.09*** | (0.03) |  |
| Backward Classes |  |  | 0.06** | (0.03) |  |
| Other Category |  |  | 0.12*** | (0.03) |  |
| Intraclass correlations |  |  |  |  |  |
| Community | 6.00%  43.97%  160.51*** | |  | 5.48%  43.60%  139.42*** |  |
| Household \| community |  |  |  |  |  |
| LR^1^ test vs linear model chi2(2) |  |  |  |  |  |
| n | 5523 | | 5523 | |  |

*^1^ Likelihood ratio*

*^2^ Standard error*

*^3^. * p<0.1, ** p<0.05, *** p<0.01*

Supplementary Table 3. Association of persistence of food insecurity and children's PPVT scores - Young Lives dataset for years 2009, 2013 and 2016.

|  | Model 1 | | Model 2 | |
| --- | --- | --- | --- | --- |
|  | Coefficient | Se^2^ | Coefficient | Se |
| Persistence of food insecurity | -0.22***^3^ | (0.04) | -0.15*** | (0.04) |
| **Children’s characteristics** |  |  |  |  |
| *Child sex* |  |  |  |  |
| Girl | Reference |  | Reference |  |
| Boy | 0.03 | (0.02) | 0.03 | (0.02) |
| Child age in months | 0.00 | (0.00) | -0.00 | (0.00) |
| Birth order | -0.03*** | (0.01) | -0.03** | (0.01) |
| Number of siblings | -0.05*** | (0.01) | -0.05*** | (0.01) |
| Child attends in private school | 0.13*** | (0.03) | 0.07** | (0.03) |
| Base PPVT score | 0.46*** | (0.01) | 0.45*** | (0.01) |
| **Household characteristics** |  |  |  |  |
| Household head age(years) |  |  | -0.00 | (0.00) |
| *Household head completed primary education* |  |  |  |  |
| No |  |  | Reference | Reference |
| Yes |  |  | 0.11*** | (0.03) |
| *Household residence* |  |  |  |  |
| Rural |  |  | Reference | Reference |
| Urban |  |  | -0.09** | (0.04) |
| *Household wealth status* |  |  |  |  |
| Lowest wealth tercile |  |  | Reference |  |
| Middle wealth tercile |  |  | 0.13*** | (0.03) |
| *Top wealth tercile* |  |  | 0.22*** | (0.04) |
| Household head caste/tribe |  |  |  |  |
| Scheduled Caste |  |  | Reference |  |
| Scheduled Tribe |  |  | -0.08* | (0.04) |
| Backward Classes |  |  | -0.04 | (0.03) |
| Other Category |  |  | -0.03 | (0.04) |
| Intraclass correlations |  |  |  |  |
| Community | 7.90%  16.70%  175.99*** | |  | 7.34%  16.22%  158.13*** |
| Household \| community |  |  |  |  |
| LR^1^ test vs linear model chi2(2) |  |  |  |  |
| n | 5610 | | 5610 | |

*^1^ Likelihood ratio*

*^2^ Standard error*

*^3^. * p<0.1, ** p<0.05, *** p<0.01*

Supplementary Table 4. Association of severity of food insecurity and children's years of completed education - Young Lives dataset for years 2009, 2013 and 2016.

|  | Model 1 | | Model 2 | |
| --- | --- | --- | --- | --- |
|  | Coefficient | Se^2^ | Coefficient | Se |
| **Severity of food insecurity** |  |  |  |  |
| Food secure | Reference |  | Reference |  |
| Mild food insecure | -0.30***^3^ | (0.05) | -0.25*** | (0.05) |
| Moderate/severe food insecure | -0.30*** | (0.05) | -0.22*** | (0.05) |
| **Children’s characteristics** |  |  |  |  |
| *Child sex* |  |  |  |  |
| Girl | Reference |  | Reference |  |
| Boy | -0.26*** | (0.04) | -0.26*** | (0.04) |
| Child age in months | 0.07*** | (0.00) | 0.07*** | (0.00) |
| Birth order | -0.07*** | (0.02) | -0.07*** | (0.02) |
| Number of siblings | -0.05** | (0.02) | -0.04* | (0.02) |
| Child attends in private school | -0.11*** | (0.04) | -0.25*** | (0.05) |
| Base education | 0.02 | (0.01) | 0.01 | (0.01) |
| **Household characteristics** |  |  |  |  |
| Household head age(years) |  |  | 0.00 | (0.00) |
| *Household head completed primary education* |  |  |  |  |
| No |  |  | Reference |  |
| Yes |  |  | 0.16*** | (0.04) |
| *Household residence* |  |  |  |  |
| Rural |  |  | Reference |  |
| Urban |  |  | -0.08 | (0.06) |
| *Household wealth status* |  |  |  |  |
| Lowest wealth tercile |  |  | Reference |  |
| Middle wealth tercile |  |  | 0.15*** | (0.05) |
| Top wealth tercile |  |  | 0.32*** | (0.06) |
| *Household head caste/tribe* |  |  |  |  |
| Scheduled Caste |  |  | Reference |  |
| Scheduled Tribe |  |  | -0.25*** | (0.07) |
| Backward Classes |  |  | -0.04 | (0.06) |
| Other Category |  |  | -0.01 | (0.07) |
| Intraclass correlations |  |  |  |  |
| Community | 8.02%  48.62%  219.07*** | | 6.78%  48.85%  177.65*** | |
| Household \| community |  |  |  |  |
| LR^1^ test vs linear model chi2(2) |  |  |  |  |
| n | 5588 | | 5588 | |

*^1^ Likelihood ratio*

*^2^ Standard error*

*^3^. * p<0.1, ** p<0.05, *** p<0.01*

Supplementary Table 5. Association of severity of food insecurity and children’s maths score- Young Lives dataset for years 2009, 2013 and 2016.

|  | Model 1 | | Model 2 | |  |
| --- | --- | --- | --- | --- | --- |
|  | Coefficient | Se^2^ | Coefficient | Se |  |
| **Severity of food insecurity** |  |  |  |  |  |
| Food secure | Reference |  | Reference |  |  |
| Mild food insecure | -0.06***^3^ | (0.02) | -0.03 | (0.02) |  |
| Moderate/severe food insecure | -0.19*** | (0.02) | -0.13*** | (0.03) |  |
| **Children’s characteristics** |  |  |  |  |  |
| *Child sex* |  |  |  |  |  |
| Girl | Reference |  | Reference | 0.00 |  |
| Boy | 0.03* | (0.02) | 0.03* | (0.02) |  |
| Child age in months | -0.00 | (0.00) | -0.00** | (0.00) |  |
| Birth order | -0.03*** | (0.01) | -0.03*** | (0.01) |  |
| Number of siblings | -0.01 | (0.01) | -0.01 | (0.01) |  |
| Private school | 0.19*** | (0.02) | 0.12*** | (0.02) |  |
| Base maths score | 0.68*** | (0.01) | 0.66*** | (0.01) |  |
| **Household characteristics** |  |  |  |  |  |
| Household head age(years) |  |  | 0.00 | (0.00) |  |
| *Household head completed primary education* |  |  |  |  |  |
| No |  |  | Reference |  |  |
| Yes |  |  | 0.13*** | (0.02) |  |
| *Household residence* |  |  |  |  |  |
| Rural |  |  | Reference |  |  |
| Urban |  |  | -0.07** | (0.03) |  |
| *Household wealth status* |  |  |  |  |  |
| Lowest wealth tercile |  |  | Reference |  |  |
| Middle wealth tercile |  |  | 0.04 | (0.02) |  |
| Top wealth tercile |  |  | 0.15*** | (0.03) |  |
| *Household head caste/tribe* |  |  |  |  |  |
| Scheduled Caste |  |  | Reference |  |  |
| Scheduled Tribe |  |  | 0.10*** | (0.03) |  |
| Backward Classes |  |  | 0.07** | (0.03) |  |
| Other Category |  |  | 0.12*** | (0.03) |  |
| Intraclass correlations |  |  |  |  |  |
| Community | 7.89%  17.26%  173.72*** | |  | 7.29% |  |
| Household \| community |  |  |  | 16.60% |  |
| LR^1^ test vs linear model chi2(2) |  |  |  | 154.36*** |  |
| n | 5519 | | 5519 | |  |

*^1^ Likelihood ratio*

*^2^ Standard error*

*^3^. * p<0.1, ** p<0.05, *** p<0.01*

Supplementary Table 6 Association of severity of food insecurity and children's PPVT scores- Young Lives dataset for years 2009, 2013 and 2016.

|  | Model 1 | | Model 2 | |
| --- | --- | --- | --- | --- |
|  | Coefficient | Se^2^ | Coefficient | Se |
| **Severity of food insecurity** |  |  |  |  |
| Food secure | Reference |  | Reference | 0.00 |
| Mild food insecure | -0.02 | (0.03) | 0.01 | (0.03) |
| Moderate/severe food insecure | -0.19***^3^ | (0.03) | -0.13*** | (0.03) |
| **Children’s characteristics** |  |  |  |  |
| *Child sex* |  |  |  |  |
| Girl | 0.00 | 0.00 | 0.00 | 0.00 |
| Boy | 0.03 | (0.02) | 0.03 | (0.02) |
| Child age in months | 0.00 | (0.00) | -0.00 | (0.00) |
| Birth order | -0.03*** | (0.01) | -0.03** | (0.01) |
| Number of siblings | -0.05*** | (0.01) | -0.05*** | (0.01) |
| Private school | 0.14*** | (0.03) | 0.07** | (0.03) |
| Base PPVT score | 0.46*** | (0.01) | 0.45*** | (0.01) |
| **Household characteristics** |  |  |  |  |
| Household head age(years) |  |  | -0.00 | (0.00) |
| *Household head completed primary*  *education* |  |  |  |  |
| No |  |  | Reference |  |
| Yes |  |  | 0.11*** | (0.03) |
| *Household residence* |  |  |  |  |
| Rural |  |  | Reference |  |
| Urban |  |  | -0.08** | (0.04) |
| *Household wealth status* |  |  |  |  |
| Lowest wealth tercile |  |  | Reference |  |
| Middle wealth tercile |  |  | 0.13*** | (0.03) |
| Top wealth tercile |  |  | 0.22*** | (0.04) |
| *Household head caste/tribe* |  |  |  |  |
| Scheduled Caste |  |  | Reference |  |
| Scheduled Tribe |  |  | -0.07* | (0.04) |
| Backward Classes |  |  | -0.03 | (0.03) |
| Other Category |  |  | -0.02 | (0.04) |
| Intraclass correlations |  |  |  |  |
| Community | 7.89%  17.26%  173.72*** | |  | 7.29% |
| Household \| community |  |  |  | 16.60% |
| LR^1^ test vs linear model chi2(2) |  |  |  | 154.36*** |
| n | 5606 | | 5606 | |

*^1^ Likelihood ratio*

*^2^ Standard error*

*^3^. * p<0.1, ** p<0.05, *** p<0.01*

**Appendix 3. Robustness extension**

Supplementary Table 7. Association of food insecurity and children's years of completed education - Young Lives dataset for years 2009, 2013 and 2016.

|  | Model 1 | | Model 2 | |
| --- | --- | --- | --- | --- |
|  | Coefficient | Se^2^ | Coefficient | Se |
| **Household is food insecure** |  |  |  |  |
| No | Reference |  | Reference | 0.00 |
| Yes | -0.15***^3^ | (0.05) | -0.08* | (0.05) |
| **Children’s characteristics** |  |  |  |  |
| *Child sex* |  |  |  |  |
| Girl | Reference |  | Reference |  |
| Boy | -0.27*** | (0.04) | -0.26*** | (0.04) |
| Child age in months | 0.07*** | (0.00) | 0.07*** | (0.00) |
| Birth order | -0.08*** | (0.02) | -0.07*** | (0.02) |
| Number of siblings | -0.05** | (0.02) | -0.04* | (0.02) |
| Private school | -0.08* | (0.04) | -0.25*** | (0.05) |
| Base education | 0.02 | (0.01) | 0.01 | (0.01) |
| **Household characteristics** |  |  |  |  |
| Household head age(years) |  |  | 0.00 | (0.00) |
| *Household head completed primary education* |  |  |  |  |
| No |  |  | Reference |  |
| Yes |  |  | 0.17*** | (0.04) |
| *Household residence* |  |  |  |  |
| Rural |  |  | Reference |  |
| Urban |  |  | -0.08 | (0.06) |
| *Household wealth status* |  |  |  |  |
| Lowest wealth tercile |  |  | Reference |  |
| Middle wealth tercile |  |  | 0.17*** | (0.05) |
| Top wealth tercile |  |  | 0.38*** | (0.06) |
| *Household head caste/tribe* |  |  |  |  |
| Scheduled Caste |  |  | Reference |  |
| Scheduled Tribe |  |  | -0.23*** | (0.07) |
| Backward Classes |  |  | -0.04 | (0.06) |
| Other Category |  |  | -0.01 | (0.07) |
| Intraclass correlations |  |  |  |  |
| Community | 8.32%  47.98%  225.91*** | | 6.94%  48.45%  180.12*** | |
| Household \| community |  |  |  |  |
| LR^1^ test vs linear model chi2(2) |  |  |  |  |
| n | 5588 | | 5588 | |

*^1^ Likelihood ratio*

*^2^ Standard error*

*^3^. * p<0.1, ** p<0.05, *** p<0.01*

Supplementary Table 8. Association of food insecurity and children's maths scores - Young Lives dataset for years 2009, 2013 and 2016.

|  | Model 1 | | Model 2 | |
| --- | --- | --- | --- | --- |
|  | Coefficient | Se^2^ | Coefficient | Se |
| **Household is food insecure** |  |  |  |  |
| No | Reference |  | Reference |  |
| Yes | -0.16***^3^ | (0.02) | -0.12*** | (0.02) |
| **Children’s characteristics** |  |  |  |  |
| *Child sex* |  |  |  |  |
| Girl | Reference |  | Reference |  |
| Boy | 0.03 | (0.02) | 0.03* | (0.02) |
| Child age in months | 0.00 | (0.00) | -0.00* | (0.00) |
| Birth order | -0.03*** | (0.01) | -0.03*** | (0.01) |
| Number of siblings | -0.01 | (0.01) | -0.01 | (0.01) |
| Private school | 0.20*** | (0.02) | 0.12*** | (0.02) |
| Base maths score | 0.68*** | (0.01) | 0.66*** | (0.01) |
| **Household characteristics** |  |  |  |  |
| Household head age(years) |  |  | 0.00 | (0.00) |
| *Household head completed primary education* |  |  |  |  |
| No |  |  | Reference |  |
| Yes |  |  | 0.13*** | (0.02) |
| *Household residence* |  |  |  |  |
| Rural |  |  | Reference |  |
| Urban |  |  | -0.07** | (0.03) |
| *Household wealth status* |  |  |  |  |
| Lowest wealth tercile |  |  | Reference |  |
| Middle wealth tercile |  |  | 0.04 | (0.02) |
| Top wealth tercile |  |  | 0.16*** | (0.03) |
| *Household head caste/tribe* |  |  |  |  |
| Scheduled Caste |  |  | Reference |  |
| Scheduled Tribe |  |  | 0.10*** | (0.03) |
| Backward Classes |  |  | 0.07** | (0.03) |
| Other Category |  |  | 0.12*** | (0.03) |
| Intraclass correlations |  |  |  |  |
| Community | 5.92%  44.81%  157.31*** | |  | 5.38% |
| Household \| community |  |  |  | 44.25% |
| LR^1^ test vs linear model chi2(2) |  |  |  | 135.88*** |
| n | 5519 | | 5519 | |

*^1^ Likelihood ratio*

*^2^ Standard error*

*^3^. * p<0.1, ** p<0.05, *** p<0.01*

Supplementary Table 9. Association of food insecurity and children's PPVT score - Young Lives dataset for years 2009, 2013 and 2016.

|  | Model 1 | | Model 2 | |
| --- | --- | --- | --- | --- |
|  | Coefficient | Se^2^ | Coefficient | Se |
| **Household is food insecure** |  |  |  |  |
| No | Reference |  | Reference | 0.00 |
| Yes | -0.18***^3^ | (0.03) | -0.14*** | (0.03) |
| **Children’s characteristics** |  |  |  |  |
| *Child sex* |  |  |  |  |
| Girl | Reference |  | Reference |  |
| Boy | 0.02 | (0.02) | 0.03 | (0.02) |
| Child age in months | 0.00 | (0.00) | -0.00* | (0.00) |
| Birth order | -0.03*** | (0.01) | -0.03** | (0.01) |
| Number of siblings | -0.05*** | (0.01) | -0.05*** | (0.01) |
| Private school | 0.14*** | (0.03) | 0.07** | (0.03) |
| Base PPVT score | 0.46*** | (0.01) | 0.45*** | (0.01) |
| **Household characteristics** |  |  |  |  |
| Household head age(years) |  |  | -0.00 | (0.00) |
| *Household head completed primary education* |  |  |  |  |
| No |  |  | Reference |  |
| Yes |  |  | 0.11*** | (0.03) |
| *Household residence* |  |  |  |  |
| Rural |  |  | Reference |  |
| Urban |  |  | -0.08** | (0.04) |
| *Household wealth status* |  |  |  |  |
| Lowest wealth tercile |  |  | Reference |  |
| Middle wealth tercile |  |  | 0.13*** | (0.03) |
| Top wealth tercile |  |  | 0.21*** | (0.04) |
| *Household head caste/tribe* |  |  |  |  |
| Scheduled Caste |  |  | Reference |  |
| Scheduled Tribe |  |  | -0.08* | (0.04) |
| Backward Classes |  |  | -0.03 | (0.03) |
| Other Category |  |  | -0.02 | (0.04) |
| Intraclass correlations |  |  |  |  |
| Community |  | 7.90% |  | 7.30% |
| Household \| community |  | 17.42% |  | 16.52% |
| LR^1^ test vs linear model chi2(2) |  | 173.37*** |  | 154.93*** |
| n | 5606 | | 5606 | |

*^1^ Likelihood ratio*

*^2^ Standard error*

*^3^. * p<0.1, ** p<0.05, *** p<0.01*

**Appendix 4. Exploratory plots**

Supplementary Figure 1. Trajectory of severe food insecurity between round 3 (2019) and round 5 (2016)


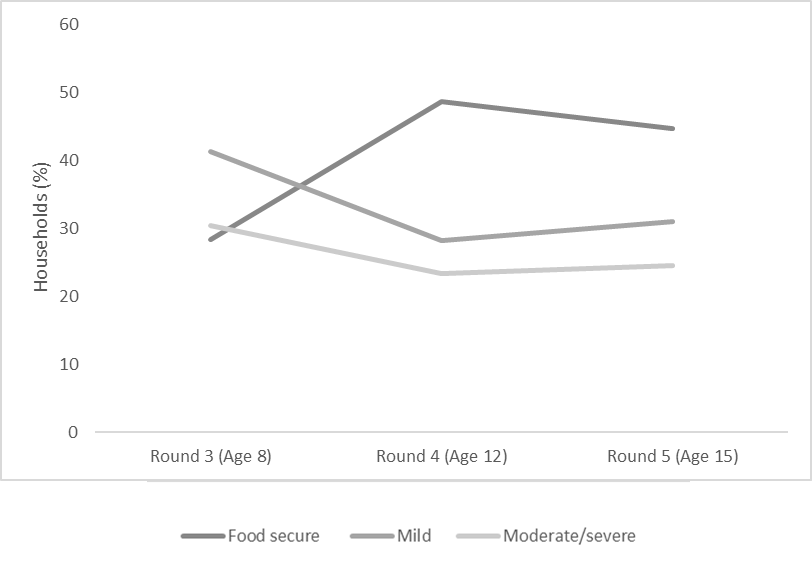


Supplementary Figure 2. Trajectory of years of education completed over rounds by severity of food insecurity categories


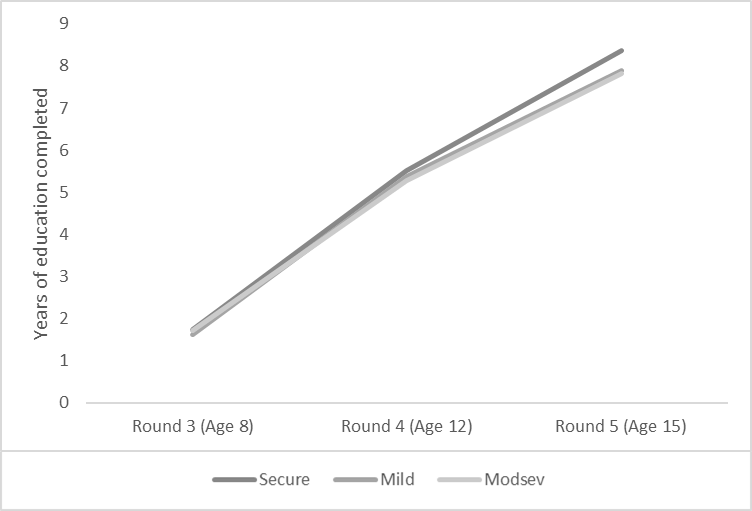


Supplementary Figure 3. Trajectory of raw maths scores over rounds by severity of food insecurity categories


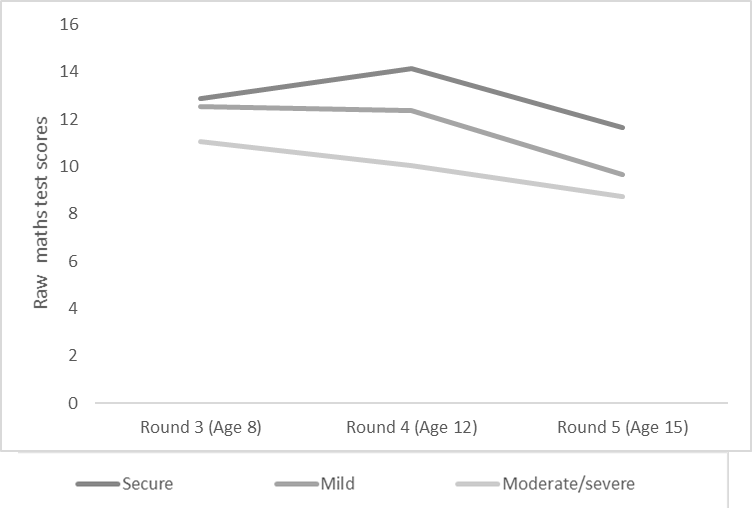


Supplementary Figure 4. Trajectory of raw PPVT scores over rounds by severity of food insecurity categories


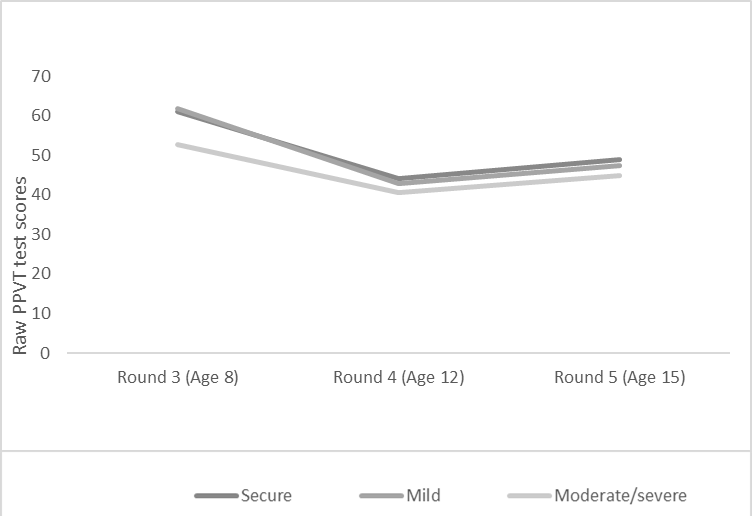

Supplement: Multimedia component1 [file mmc1.docx]
